# Blood Pressure and Chronic Kidney Disease Progression: An Updated Review

**DOI:** 10.7759/cureus.24244

**Published:** 2022-04-18

**Authors:** Elmukhtar Habas, Eshrak Habas, Fahmi Y Khan, Amnna Rayani, Aml Habas, Mehdi Errayes, Khalifa L Farfar, Abdel-Naser Y Elzouki

**Affiliations:** 1 Internal Medicine, Hamad Medical Corporation, Doha, QAT; 2 Internal Medicine, Hamad General Hospital, Doha, QAT; 3 Internal Medicine, Tripoli University, Tripoli, LBY; 4 Hemato-oncology Department, Tripoli Pediatric Hospital, Tripoli University, Tripoli, LBY; 5 Renal and Dialysis Department, Tripoli Pediatric Hospital, Tripoli, LBY; 6 Internal Medicine, Alwakra Hospital, Alwakra, QAT

**Keywords:** htn, bp record, bp diurnal variation, home blood pressure, ckd progression

## Abstract

Hypertension (HTN) is common in chronic kidney disease (CKD), and it may aggravate CKD progression. The optimal blood pressure (BP) value in CKD patients is not established yet, although systolic BP ≤130 mmHg is acceptable as a target. Continuous BP monitoring is essential to detect the different variants of high BP and monitor the treatment response. Various methods of BP measurement in the clinic office and at home are currently used. One of these methods is ambulatory BP monitoring (ABPM), by which BP can be closely assessed for even diurnal changes.

We conducted a non-systematic literature review to explore and update the association between high BP and the course of CKD and to review various BP monitoring methods to determine the optimal method for BP recording in CKD patients. PubMed, EMBASE, Google, Google Scholar, and Web Science were searched for published reviews and original articles on BP and CKD by using various phrases and keywords such as "hypertension and CKD", "CKD progression and hypertension", "CKD stage and hypertension", "BP control in CKD", "BP measurement methods", "diurnal BP variation effect on CKD progression", and "types of hypertension." We evaluated and discussed published articles relevant to the review objective. Before preparing the final draft of this article, each author was assigned a section of the topic to read, research deeply, and write a summary about the assigned section. Then a summary of each author's contribution was collected and discussed in several group sessions.

Early detection of high BP is essential to prevent CKD development and progression. Although the latest Kidney Disease Improving Global Outcomes (KDIGO) guidelines suggest that a systolic BP ≤120 mmHg is the target toprevent CKD progression, systolic BP ≤130 mmHg is universally recommended.ABPM is a promising method to diagnose and follow up on BP control; however, the high cost of the new devices and patient unfamiliarity with them have proven to be major disadvantages with regard to this method.

## Introduction and background

Chronic kidney disease (CKD) is a global health problem, with an estimated prevalence of 11-13% [1], and it is continuously on the rise [2]. CKD can progress to end-stage renal disease (ESRD) in rare cases [3] as well as cardiovascular disease (CVD), leading to high rates of mortality [4]. CKD prevention and reducing its progression rate are major concerns in healthcare settings.

The prevalence of hypertension (HTN) in CKD patients is 80-85%, which is significantly higher than in the general population (28.5%) [5,6]. This difference in HTN prevalence is attributed to an increase in the aging population [7] as well as the prevalence of diabetes mellitus [8]. Blood pressure (BP) changes are associated with alteration of kidney function, and high BP worsens CKD, thereby increasing ESRD incidence and CVD risk [9,10].

Optimum BP control is essential in CKD patients to prevent CKD progression and other complications [11]. HTN prevalence is inversely related to the estimated glomerular filtration rate (eGFR), increasing significantly from 67% in patients with an eGFR of 60 ml/min/1.73 m^2^ to 92% in those with an eGFR of 30 ml/min/1.73 m^2^ [12,13]. The variability of 24-hour BP in CKD patients is higher than that in the general population [14]. In addition, a mean systolic BP >120 mmHg is associated with imminent CKD and increases the rate of sudden renal function deterioration [9]. Studies have reported that lowering BP reduces CKD progression and CVD risks; however, the ideal BP value in CKD patients has yet to be established [15,16]. The most recent guidelines state that a BP <120 mmHg as the lowest targeted is desirable in CKD patients; nevertheless, the targeted BP in CKD patients differs between various published guidelines [11,17]. Furthermore, it has been recently reported that intensive BP control may reduce eGFR and increase tubule biomarkers [18].

Mercury-based sphygmomanometer devices are the primary BP measuring techniques currently used. However, automatic, ambulatory BP monitoring (ABPM), and ultrasonic-based methods have recently gained currency as BP recording techniques. Each technique has its own limitations, advantages, disadvantages, and abilities to detect BP variations and assess the response to treatment. In light of this, we aim to discuss and update the effect of BP on CKD progression and explore the various methods of BP recording in CKD patients.

## Review

CKD and HTN

CKD is classified into five main stages and further subdivided into subgroups according to the GFR value and albuminuria [19]. There is reportedly an evident association between the interstitial fibrosis and tubular atrophy and the mean 24-hour ABPM records [20]. There is substantial evidence to indicate that CKD is strongly linked to uncontrolled high BP, resistant HTN, masked HTN, and even normotensive non-dipping BP records [21].

In their study, Aggarwal et al. found that in non-tight glycemic control diabetic patients with CKD stage 3 or more, intensive systolic BP management (130 mmHg) lowers all-death causes when compared to a BP of 140 mmHg [22]. Another recent study has reported a significant improvement in renal outcomes in patients who had diastolic BP ≥90 mmHg [23]. The same study noted that patients with BP <130/80 mmHg had the benefit of risk reduction in terms of CKD progression, even in cases that had proteinuria at presentation. Furthermore, after a year of systolic BP control (<130 mmHg), the risk of CKD progression decreased by 42% in those with systolic BP ≥130 mmHg at baseline, and their renal outcomes also improved [23]. The Systolic Blood Pressure Intervention Trial (SPRINT) concluded that tight BP control reduces mortality rates in CKD patients [15]. Appel et al., on the other hand, reported that intensive BP control had no effect on CKD progression, despite the fact that tight BP control may have different effects on patients with or without proteinuria at presentation [24]. Son et al. concluded that renal outcomes and death are related more to abnormal 24-hour ABPM than office BP records in CKD patients [25]. A Korean study has reported that advanced renal dysfunction and proteinuria were significantly related to non-dipping BP records. As per another study, in obese diabetic Chinese patients with CKD, the HTN control was suboptimal, increasing the risk of diabetic hypertensive kidney complications [26].

The International Society of Hypertension (ISH) has modified the worldwide practice guidelines for HTN diagnosis and management in adults aged ≥18 years [27]. The guidelines for HTN diagnostic criteria have been updated concisely and regularly and streamlined for universal use. According to the American College of Cardiology/American Heart Association (ACC/AHA) 2017 guidelines, lowering BP in CKD patients to a level <130/80 mmHg reduces CKD progression [11].

In CKD, careful HTN control is critical to slow the progression of the disease. The importance of BP measurement and strict BP control are emphasized in the recent Kidney Disease Improving Global Outcomes (KDIGO) guidelines on BP therapy in CKD [28]. Although the recent KDIGO guidelines recommend a systolic BP target of <120 mmHg, the recommendation has been controversial because it is based on limited data from only one randomized controlled trial and its CKD subgroup analysis [16]. According to Dasgupta et al., this target systolic BP cannot be extrapolated to routine clinic BP measurements because it may cause falls and fractures in multimorbid and frail CKD patients. Furthermore, achieving this target BP in the majority of CKD patients appears to be a challenge for both patients and physicians. Moreover, the newly recommended systolic BP in the new KDIGO guidelines is an outlier among current major international HTN guidelines, which may confuse clinicians in the future. Therefore, the new systolic BP target of <120 mmHg established by KDIGO for CKD patients may be harmful and inappropriate for the majority of the CKD patients [28].

Types of HTN

Primary (essential) and secondary HTN are the two basic types of HTN. Primary HTN accounts for the majority of cases (95%), and it has no known cause. Thyroid disorders, sleep apnea, excessive alcohol use, obesity, high-salt food consumption, tumors, adrenal hyperfunction, medicines, kidney illness, and other factors are all linked to secondary HTN. In addition to these HTN categories, other types such as isolated systolic, malignant, resistant, labile, and white-coat HTNs have also been identified, and these have their own particular diagnostic criteria.

Isolated systolic HTN is diagnosed when the systolic BP is >140 mmHg, and the diastolic BP is ≤80 mmHg. Isolated systolic HTN is more widely prevalent in people aged >65 years, and it is most probably due to arterial wall stiffness [29]. The incidence of malignant HTN type is two new cases/100,000/year in the general population [30]. It is highly prevalent among young adults, individuals with eclampsia, and African American men [30]. Malignant HTN is diagnosed when the BP increases suddenly and the diastolic BP reaches >130 mmHg and when there are bilateral retinal exudates and/or hemorrhages, with or without papilledema [31]. It is considered a medical emergency and needs prompt management. Malignant HTN is a type of HTN in which the systolic BP is ≥140 mmHg or the diastolic BP is ≥90 mmHg despite the concurrent use of three antihypertensive medications of various classes, including a diuretic, or controlled BP with more than four antihypertensive medications [11]. Resistant HTN has been reported in 20-30% of hypertensive cases. Resistant HTN appears to have an underlying genetic component. It is commonly seen in the elderly, obese women, African Americans, diabetics, and CKD patients.

White-coat and labile HTN are types of HTN that are not uncommon. These terms have been alternatively used to label cases with high normal BP levels. These cases are more likely to have HTN, although they may indicate an increase in BP as a physiological response. Labile HTN occurs in almost every individual, and it can be related to BP record changes due to factors such as stress, environment, food, etc. On the other hand, a white-coat HTN diagnosis is usually given to subjects who have high BP levels when measured by a medical professional but have normal BP levels when recorded in other settings. Therefore, due to the variability of BP values and the effect of white-coat, the American Heart Association recommends that BP measurement must be conducted a minimum of three times during different times of the day for a precise HTN diagnosis. However, some physicians prefer to reassess BP 5-10 minutes after the first high BP record in the same setting in a single visit. White-coat HTN was reported in 28.8%, and masked HTN was noted in 7% of CKD patients in one study [32], causing CKD and affecting its progression.

Night-time HTN and non-dipping night BP patterns are the other types that may negatively affect end organs. Non-dipping is not a specific independent risk factor that negatively impacts CKD outcomes, although it is a recognized risk factor for CKD progression. However, the early studies on the subject were not controlled for proteinuria. Proteinuria has been linked to BP non-dipping, and proteinuria is a risk factor for CKD deterioration [33]. As a result, it was proposed that the non-dipping BP effect on CKD development is doubtful, but instead, non-dipping is a pathological process that may increase CKD deterioration. Furthermore, when non-dipping was examined as a risk factor for mortality, ESRD, and CVD events, it was found to be insignificant when daytime BP or 24-hour BP were considered and CVD event risk factors were excluded [33,34]. However, according to one study, non-dipping is still a significant risk factor for both ESRD and CVD events, even after controlling for BP, when it was assessed by 24-hour monitoring, in the presence of proteinuria [35]. There is enough evidence to show that all phenotypes of HTN occur in CKD patients, thereby affecting CKD development and progression.

HTN is a well-known cause of CKD, and CKD is also a cause of secondary HTN. HTN associated with CKD causes is multi-factorial. Sodium dysregulation [36], decreased kidney tissue mass and function [37], increased sympathetic system activity, and distributed renin-angiotensin-aldosterone system function [38] are the possible mechanisms of HTN in CKD patients. Advanced CKD (eGFR of 30 mL/min/1.73 m^2^) is characterized by endothelial dysfunction, and these endothelial alterations are strongly related to HTN [39]. Additionally, CKD is associated with an increased rate of arterial stiffness [40], implying the development of HTN [41]. As HTN progresses, variables such as increased oxidative metabolism associated with renal hypoxia may contribute to elevated BP and CKD progression [42,43]. Detection of these phenotypes is not usually possible with the clinic office BP measurement methods, but these are easily detected with ABPM. It appears that ABPM is the best method to differentiate between various HTN phenotypes and also to assess their response to therapy.

General considerations for high-BP management

The number of hypertensive patients aged between 30 and 79 years has doubled in the recent decades, increasing from an estimated 317 million men and 331 million women in 1990 to about 626 million women and 652 million men in 2019. More than 50% of these hypertensive individuals (720 million) received no treatment in 2019 [44]. Globally, only 23% and 18% of women and men had well-controlled BP respectively. The increase in the HTN rate is mainly due to the increase in the number of aging populations worldwide. The rising number of hypertensive population has not shown a uniform pattern, and HTN prevalence is higher in most low- and middle-income countries. In high-income countries, counterweighing progress in HTN prevalence reduction has been noted. It has been claimed that the great success in early HTN detection and treatment in developed countries has indicated the possibility of reducing HTN prevalence and complications in middle- and low-income countries as well [45].

Setting optimal BP targets has a sizeable clinical impact [11] on morbidity, mortality, and renal function outcomes. According to the published guidelines, systolic BP of 130 mmHg is categorized as stage 1 HTN. Non-CKD patients with this level of BP have been advised to change their eating and exercise habits to control their BP. The reports on HTN management among CKD patients have been inconclusive. Studies have revealed that a targeted BP <130/80 mmHg, as compared to a BP <140/90 mmHg, is useful in decelerating renal disease progression in proteinuric CKD patients; however, other studies have not endorsed these suggestions [36]. A significant reduction in mortality rate after intensive BP control in CKD patients has not been consistently validated in RCTs, as other trials were not powered enough to investigate this outcome, and the supporting evidence was primarily obtained from observational studies [46,47]. However, a study by Ku et al. has suggested that strict BP control has no effect on delaying ESRD onset, even though it may decrease the risk of mortality in CKD cases [48]. There is evidence to suggest that the advocated mortality benefit could depend on proteinuria existence at presentation; however, this assumption was also derived from observational trials [36,49]. Additionally, the incidence of CVD outcomes in CKD patients who were adherent to intensive targets was poorly characterized [15].

BP measurement methods and recommendations

BP measurement is usually conducted by a doctor or nurse with a sphygmomanometer in their clinics; however, the recent wide availability of semi or fully automatic machines has enabled medical professionals or patients themselves to measure BP. Currently, there are four methods to measure BP.

A. Clinic Office BP Measurement Methods

Clinic office BP recording needs to fulfill specific requirements, which are summarized in Table 1 [50].

**Table 1:** BP measurement requirements and conditions BP: blood pressure

Factors	Description
BP measurement place	Office of the physician or the nurse
Condition of place	A quiet room with a temperature between 36.5–37.5 ^o^C
Patient status	Before BP recording: no cigarette smoking, no caffeine consumption, and no exercise (even walking) for 30 minutes; empty bladder; should remain seated and relaxed for at least 3–5 minutes
Machine	The machine must be validated, especially the oscillometric electronic machine, regularly. Validated accurate electronic devices for use in office and home, and ambulatory BP measurement devices for adults and pregnant women are available
Patient and the personnel	Patients and staff should not communicate before, during, and between measurements. The patient must be sitting, and the arm must be rested on a table at the level of the heart. The patient's back should be supported on a chair. The patient's feet must be in a flat position, and legs should be uncrossed
Cuff and balloon	Cuff and its balloon should be selected according to the individual’s arm circumference. A manual auscultatory inflatable balloon should cover 75–100% of the arm circumference. For the cuffs of electronic devices, see the instruction booklet that comes with the device
BP measurement protocol	Three records must be taken a minute apart during each visit, and a mean of the last two stable records is then calculated. If the first BP record is <130/85 mmHg, no more re-measurements are needed. If 2-3 office-based records show BP ≥140/90 mmHg, it indicates hypertension

1. Auscultatory method: this method is the gold standard for clinic office BP recording. This mercury-based device used to measure BP is currently sparingly used and even abandoned in various countries due to the toxicity risk associated with mercury [51]. New hybrid incorporated sphygmomanometers are rapidly replacing the old version of mercury-based devices. The mercury-based sphygmomanometer method requires a medical professional to detect Korotkoff sounds and record BP, which may adversely affect BP records [51]. Automatic and aneroid BP record devices are more practical, and people can use them independently, even in the clinic; however, their lack of precision and the need for regular calibration limit their use [51].

2. Automated office BP monitoring: novel non-mercury-based oscillometric devices are now widely used for BP recording in the clinic office [52], and other sites such as pharmacies, homes, and work offices. These devices are either semi-automatic or fully automatic, which can sense the changes in the blood flow and the artery wall that refect the BP record, thereby reducing BP variability due to the presence of medical staff, although it may increase patient BP reporting bias.

B. Home Self-BP Monitoring Methods

1. Intermittent home self-BP measurement method: intermittent BP monitoring by fully automatic electronic devices at home is widely practiced in developed countries to confirm HTN and follow up on the response to treatment. Furthermore, the intermittent home BP recording method is a more suitable and acceptable alternative to the ABPM method to differentiate between HTN phenotypes [53]. Higher intermittent home BP values are associated with CKD progression compared to clinic office-based BP records [54]. However, the high cost of the device, patient unfamiliarity, and inaccurately reported records by the subjects have made the home BP monitoring method not very ideal. A home-clinic BP telemonitoring system has been invented to minimize patient BP reporting bias, especially in HTN control follow-ups. Recently, it was reported that the zero digits in the electronic device have reduced the chance of bias and inaccurate patient reporting [55].

2. ABPM method: ABPM has been in use for more than 30 years. The ABPM technique is used to measure BP every 15 minutes during the day and every 30 minutes during the night while subjects are conducting their daily activities [27]. One of the advantages of ABPM is BP measurement at night, which can detect the night BP pattern during sleep. Low eGFR is reported in masked, true sustained, night-time HTN, and normal BP non-dipping patterns at night [21].

It has been noted that abnormal ABPM pattern is typically related to proteinuria than to deterioration of eGFR in CKD patients [56]. Also, it has been reported that arterial stiffness is increased in uncontrolled masked BP variant, leading to continuous progressive eGFR reduction and BP increase [57]. It was claimed that frequent abnormal ABPM records are linked to vascular endothelium dysfunction, increased sympathetic tone, sodium retention, and induced inflammatory reactions [58]. The recommended BP records in both office and ABPM methods to diagnose HTN are presented in Table 2.

**Table 2:** Hypertensive record limits in office and ambulatory BP monitoring (ABPM) BP: blood pressure

Place of BP measurement	Systolic/diastolic BP records (mmHg)
Hypertension office-based BP record	≥140/≥90
Ambulatory BP monitoring average record	
Hypertension average 24-hour record	≥130/≥80
Hypertension awake time (daytime)	≥135/≥85
Hypertension night-time (sleeping)	≥120/≥70

C. Central BP Monitoring Method

Central BP monitoring is an invasive BP recording technique. This technique is performed by inserting an arterial catheter with tonometry at the carotid artery [59]. Although the internal kidney pressure is correspondent to the central than peripheral BP, the relationship between the central BP and kidney function assessed by urine protein and eGFR is not well investigated. Hence, further studies are required to assess the relationship between central BP and the degree of eGFR, proteinuria, and CKD progression.

D. Smartphone and Other Device-Based BP Monitoring Method

The smartphone-based method of BP recording is a recently invented way to measure BP via smartphone applications [60,61]. This method has been reported to be inaccurate in BP measurement. Even though phone-, watch-, or scale-based devices can record the peripheral BP, they are not currently able to read the brachial BP. There is not enough data supporting their usage in continuous BP monitoring as ABPM except for one reported study involving an iPhone-based application. To the best of our knowledge, their accuracy and BP records have not been assessed in CKD patients.

Recently, Degott et al., based on their analysis of 353 paired recordings from 91 persons, have reported that "the smartphone embedded OptiBP cuffless mobile application achieves the validation requirements of the Association for the Advancement of Medical Instrumentation (AAMI), the European Society of Hypertension (ESH), and the International Organization for Standardization (ISO), universal standards in a general population for the measurement of diastolic BP and systolic BP" [62]. Another study conducted by Chandrasekhar et al. concluded that the oscillometric finger-pressing method for measuring BP on a smartphone demonstrated bias and precision errors in systolic and diastolic BP records. These inaccuracies were equivalent to those observed when using the finger-cuff device. The same study noted that cuff-less and calibration-free systolic and diastolic BP monitoring can be possible with a smartphone-based method [63].

Recommendations based on current guidelines

In November 2015, the U.S. Preventive Services Task Force (USPSTF) accepted the clinic office's repeated BP recording method to diagnose HTN and advised that BP be recorded outside of the clinic office to confirm HTN before treatment and follow-up [53]. The USPSTF advice was based on the fact that 25% of people who had high BP in the clinic office setting had reportedly turned normotensive when measured by the ABPM method and would not require treatment. The Eighth Joint National Committee and the European Society of HTN (ESH) advised that office BP is the gold standard for HTN diagnosis and treatment [61]. On the other hand, ESH 2013, ACC/AHA 2017, and ISH 2020 guidelines have advised using intermittent home or ABPM methods if the white-coat and masked HTN are suspected [27]. None of the published guidelines have reported that the central BP recoding technique can be used for routine HTN diagnosis or treatment follow-up [58]. The recommended BP recording methods by the existing guidelines are summarized in Table 3.

**Table 3:** Recommended BP recording methods as per the existing guidelines BP: blood pressure

Guidelines	Recommended BP recording methods
European Society of Hypertension (ESH), 2013	Intermittent home or ambulatory BP monitoring if the white-coat and masked HTN are suspected
8th Joint National Committee (JNC), 2014	Office BP recording
U.S. Preventive Services Task Force (USPSTF), 2015	Repeated office BP measurements with BP recorded outside the clinic office
American College of Cardiology (ACC)/American Heart Association (AHA), 2017	Intermittent home or ambulatory BP monitoring if the white-coat and masked HTN are suspected
International Society of Hypertension (ISH), 2020	Intermittent home or ambulatory BP monitoring if the white-coat and masked HTN are suspected

Automatic BP measuring devices with multiple records in the physician clinic are better at overcoming white-coat HTN overdiagnosis compared to the usual clinic office BP recording method [64]. Numerous automatic office-based BP records significantly correspond to the average BP records during the daytime [65], although it is not always consistent [66]. Although 2017 ACC/AHA recommendations did not specifically advocate that automatic office BP recordings are superior to ABPM, they noted that "there is growing evidence supporting the use of automated office BP" [67]. ISH 2020 guideline recommendation has not stated that ABPM is superior to the office automatic BP measurement method [27].

BP measurement by ABPM or home BP recording is highly recommended to diagnose and manage HTN in the newly published guidelines [11,15,17]. ABPM allows daytime, night-time, and 24-hour BP determination and diurnal BP variation recording. ABPM reading is strongly linked with target organ damage and CVD events than office or clinic BP readings [68], although these conclusions were drawn from studies with several limitations [69].

Gender and CKD progression

Several guidelines have suggested that early HTN detection and prompt treatment can delay the CKD progression and decrease complications in both genders [61,70]. However, the degree to which females and males with HTN are at equal risk to develop CKD outcomes was not thoroughly examined. Murphy et al. have predicted that the prevalence of CKD is more common in females [71], although Albertus et al. have noted that ESRD risk is higher in the male gender [72]. A meta-analysis review of the gender-specific effect of CKD progression on non-diabetic CKD patients proposed that females progress at a slower rate toward ESRD than males, regardless of the underlying cause [73]. On the contrary, another patient-level meta-analysis study that used angiotensin-converting enzyme inhibitors to control the BP concluded that renal disease progression rate is possibly quicker among females than males [74]. Recently, a study reported an unequal influence of HTN on CKD progression in females versus males, and the discrepancy is questionable to be related to biological differences alone [75].

## Conclusions

BP measurement is essential for diagnosis and following up on the response to therapy. Accurate diagnosis and detection of the HTN phenotype are critical to preventing CKD complications development and progression. Despite the diversity of methods and devices for BP measurement, the mercury sphygmomanometer is still considered the gold standard. However, new devices are increasingly being used currently, but they are not highly accurate and need frequent calibrations. Although it appears that ABPM is the best method to confirm or rule out HTN and differentiate between various HTN phenotypes, its superiority over the other BP measurement methods is questionable and not clearly established. Therefore, new research projects are required to assess and explore the superiority of the ABPM technique.

Clinicians should reach a consensus that a systolic BP level <130 mmHg in CKD improves all-cause mortality rates, despite the variations between the reported data. However, further studies are required to assess the link between the early detection and control of high BP levels and CKD development and progression patterns. We propose that tight control of BP at a level <130/80 mmHg is highly recommended and that ABPM is a highly reliable method for HTN diagnosis, especially in doubtful cases, and it is especially more effective in predicting CKD progression.
